# Supplementary material for: How does childhood maltreatment influence cardiovascular disease? A sequential causal mediation analysis
Source: Int J Epidemiol. 2021 May 26;51(2):555–66. doi: 10.1093/ije/dyab085 (PMC9082818; doi:10.1093/ije/dyab085)
Supplement: dyab085_Supplementary_Data [file dyab085_supplementary_data.pdf]

How does childhood maltreatment influence cardiovascular disease? A sequential causal mediation analysis in UK Biobank

SUPPLEMENTARY MATERIAL

Supplementary methods

Cardiovascular disease at baseline

Baseline cardiovascular disease (CVD) was based on self-reported medical diagnosis of CVD and hospital records of CVD events that occurred prior to baseline, which occurred between 2006-2010. Self-reported CVD was assessed using the question: “Has a doctor ever told you that you have had any of the following conditions?”, considering the options: heart attack, angina, stroke, high blood pressure, blood clot in the leg (deep vein thrombosis) and blood clot in the lung. Hospital registers date back to 1997 for England, 1998 for Wales and 1981 for Scotland, and were coded according to the International Classification of Diseases (ICD). We defined baseline CVD as self-reported CVD, or ICD-10 codes: I00-I99 or ICD-9 codes: 390-459 registered before the date of assessment.

Childhood maltreatment

Childhood maltreatment was assessed at the follow-up online mental health questionnaire using the Childhood Trauma Screener (CTS), which is a short form of the Childhood Trauma Questionnaire, with five items referring to “When I was growing up”. Items indexed: i. Physical abuse - “People in my family hit me so hard that it left me with bruises or marks”, ii. Sexual abuse - "Someone molested me (sexually)", iii. Emotional abuse - "I felt that someone in my family hated me", iv. Emotional neglect - "I felt loved", and v. Physical neglect - "There was someone to take me to the doctor if I needed it". The response options were on a 5-point Likert scale ranging from (1) never true to (5) very often true, and childhood maltreatment types were defined as present/absent according to the following cut-off points of the CTS: physical abuse: ≥1, sexual abuse: ≥1, emotional abuse: ≥1, emotional neglect: ≤2, and physical neglect: ≤3.

Mediators

Anxiety/depression was defined as a positive answer to either of the two questions: “Have you ever seen a general practitioner (GP) for nerves, anxiety, tension or depression?” or “Have you ever seen a psychiatrist for nerves, anxiety, tension or depression?”. Smoking was

considered as ever smoking (current tobacco smoking occasionally or most/all days, or past tobacco smoking most days, occasionally, or tried once or twice). BMI (kg/m^2) was calculated using weight and height measured at recruitment. Serum CRP (mg/L) was measured by immunoturbidimetric - high sensitivity analysis on a Beckman Coulter AU5800. Given the positive skewed distribution of CRP, it was log-transformed for analyses and back-transformed (exponentiated) for presentation of results.

Confounders

Information on age, year of birth, ethnicity, maternal smoking around birth, number of siblings, family history of CVD, Townsend deprivation index, and education were assessed at recruitment. Age (complete years) was calculated based on date of birth and date of attending the initial assessment centre. Year of birth was assessed in years and classified into <1950, 1950-1959 and 1960-1970. Ethnicity was classified as White, Mixed, Asian, Black, Chinese and Others. Maternal smoking around birth (yes/no) was assessed by the question: "Did your mother smoke regularly around the time when you were born?", and classified into yes, no, or do not know. Number of siblings was based on the number of participant's brothers and sisters and categorised into 0, 1, 2, 3, 4 or 5+. Family history of CVD was based on self-report of high blood pressure, stroke or heart disease of the mother, father or siblings. Townsend deprivation index was calculated based on preceding national census output areas, and each participant was assigned a score corresponding to the output area in which their residing postcode was located; the index was divided into quintiles. Educational attainment was assessed in 5 categories: College, university or other professional degree; Advance levels/ advance Subsidiary levels or equivalent; Ordinary levels/ General Certificate of Secondary Educations or equivalent; Certificate of Secondary Educations or equivalent/ National Vocational Qualifications/ Higher National Diploma, Higher National Certificate or equivalent/ other professional qualification; and None.

Sequential mediation analysis

To estimate direct and indirect effects, the following assumptions regarding confounding are needed: a) no unmeasured confounding for the exposure-outcome relationship, b) no unmeasured confounding for the mediator-outcome relationship, c) no unmeasured confounding for the exposure-mediator relationship, and d) no effects of the exposure that confounds the mediator-outcome relationship (VanderWeele & Vansteelandt, 2014). With the approached used in our study (parametric g-computation using Monte Carlo

simulations), there is therefore no need to make assumption d, which is needed when using standard methods for mediation (Daniel, 2011). We considered the natural direct effect (NDE) and natural indirect effect (NIE) as decompositions of the total causal effect (TCE); *gformula* implements logistic regression for time-to-event outcomes and therefore estimates correspond to log odds ratios (ORs). We exponentiated the results and present ORs.

To estimate the combined and individual contribution of the mediators, we performed four models using sequential mediation analysis. In Model 1, we estimated the NIE through anxiety/depression; this includes pathways that act through anxiety/depression and any of its effects (descendants) but does not include those that act solely through smoking, BMI and/or CRP (or other pathways). In Model 2, the NIE through both anxiety/depression and smoking was estimated, which includes their causal descendants, but does not include any other pathways, including paths that act only via BMI and/or CRP; the difference between the proportion mediated in Model 2 and the proportion mediated in Model 1 corresponds to the proportion mediated by smoking beyond anxiety/depression alone (i.e. the additional contribution of smoking). In Model 3, the NIE through anxiety/depression, smoking and BMI was estimated, which includes their causal descendants, but does not include the paths that act solely through CRP; the difference between the proportions mediated in Model 3 and Model 2 corresponds to the mediating effect of BMI beyond the effect of both anxiety/depression and smoking. Finally, in Model 4, the NIE of all potential mediators was estimated, and the difference between the proportion mediated in Model 4 and the proportion mediated in Model 3 corresponds to the mediating effect of CRP beyond anxiety/depression, smoking and BMI. Exposure-mediator interactions were included sequentially (i.e. interaction between maltreatment and anxiety/depression was included in Model 1, interactions between maltreatment and anxiety/depression and between maltreatment and smoking were included in Model 2, and so on). We did not include mediator-mediator interactions, as these would hinder comparability across models (VanderWeele & Vansteelandt, 2014). Bootstrapping with 1,000 replications was used to calculate 95% CIs. The number of Monte Carlo simulations was equivalent to the sample size of each model. The proportion mediated was calculated as $\frac{OR\ NDE\ x\ (OR\ NIE-1)}{(OR\ NDE\ x\ OR\ NIE-1)}\ x\ 100$.

References

Daniel RM, De Stavola BL, Cousens SN. gformula: Estimating causal effects in the presence of time-varying confounding or mediation using the g-computation formula. Stata J. 2011;11(4):479-517.

VanderWeele TJ, Vansteelandt S. Mediation analysis with multiple mediators. Epidemiol Methods. 2014;2(1):95-115.

Supplementary Table S1. Associations of childhood maltreatment, mediators and cardiovascular disease with having complete data (no missing mediators and/or confounders).

	Men		Women	
	OR (95% CI)	p-value	OR (95% CI)	p-value
Physical abuse	1.00 (0.91; 1.10)	0.988	0.98 (0.91; 1.06)	0.683
Sexual abuse	1.10 (0.93; 1.31)	0.267	0.98 (0.89; 1.08)	0.738
Emotional abuse	1.03 (0.92; 1.16)	0.608	0.95 (0.88; 1.03)	0.190
Emotional neglect	0.98 (0.90; 1.08)	0.742	0.94 (0.88; 1.01)	0.110
Physical neglect	0.88 (0.79; 0.98)	0.018	0.91 (0.84; 0.99)	0.020
Cardiovascular disease	1.04 (0.93; 1.16)	0.529	1.04 (0.94; 1.15)	0.430

Adjusted for age, year of birth, ethnicity, maternal smoking, number of siblings and family history of cardiovascular disease. Models for cardiovascular disease were further adjusted for education and Townsend deprivation index

Supplementary Table S2. Comparison of participants with no CVD at baseline who were included and not included in the analyses.

	Males		Females	
	Included<br>N = 40,596<br>(30.0%)	Not included<br>N = 94,675<br>(70.0%)	Included<br>N = 59,511<br>(32.7%)	Not included<br>N = 122,366<br>(67.3%)
Continuous variables: mean (SD)				
Age (years)	55.2 (7.9)	54.8 (8.5)	54.5 (7.6)	55.3 (8.2)
BMI (kg/m^2)	26.6 (3.6)	27.2 (3.8)	25.7 (4.5)	26.5 (4.8)
CRP (mg/L)	2.0 (3.7)	2.3 (4.1)	2.1 (3.6)	2.5 (4.1)
SBP (mmHg)	137.3 (15.8)	138.4 (16.7)	130.3 (17.1)	132.2 (18.3)
DBP (mmHg)	82.6 (9.3)	83.3 (9.6)	78.8 (9.3)	79.5 (9.6)
Categorical variables: %				
Ethnicity (White)	97.2	93.4	97.2	93.9
Townsend deprivation index				
1st quintile (20% least deprived)	22.9	19.0	21.5	19.1
5th quintile (20% most deprived)	16.2	22.2	17.0	21.0
Qualifications				
None of the below	5.6	16.7	5.7	17.6
Other/ NVQ / CSE	14.6	20.4	12.1	16.9
O-level	16.7	20.0	21.1	24.7
A-level	12.6	10.3	14.7	11.6
College / University degree	50.5	32.6	46.3	29.2
Number of siblings				
0	12.1	11.3	11.2	11.3
1	35.7	31.3	35.4	31
2	26.2	24.7	26.8	24.7
3	13.3	13.8	13.8	14
4	6.1	7.4	6.2	7.6
5+	6.5	11.5	6.7	11.5
Maternal smoking				
No	63.5	61.5	64.7	63.4
Yes	25.0	25.6	24.8	24.9
Do not know	11.5	12.8	10.5	11.7
Smoking status				
Never smoker	37.5	37.1	44.4	45.3
Former smoker	53.6	48.3	49.3	44.2
Current smoker	8.9	14.7	6.3	10.4
Alcohol intake				
Daily or almost daily	27.9	23.4	19.2	15.5
3-4 x/ week	29.5	25.9	25	20.5
1-2 x/ week	24.9	27.4	25.9	26.7
1-3 x/ month	8.5	9.3	12.8	13.2
Special occasions only	5.2	7.5	11.1	14.8
Never	4.1	6.4	6.0	9.4
Depression	23.6	24.2	38.3	40.1
Use of blood pressure medication	0.7	1.2	0.5	0.6
Family history of CVD	68.7	66.2	75.2	73.9
Cardiovascular disease	15.7	20.4	10.8	15.4

Participants not included in the analyses were those who did not respond the online mental health questionnaire with information on childhood maltreatment or had missing data on mediators and/or confounders.
A-level: advanced level or equivalent; BMI: body mass index; CSE: certificate of secondary education or equivalent; CVD: cardiovascular disease, DBP: diastolic blood pressure; HND: higher national diploma of equivalent; NVQ: national vocational qualification, O-level: ordinary level; SBP: systolic blood pressure; SD: standard deviation

Supplementary Table S3. Unadjusted and adjusted association between childhood maltreatment and incident cardiovascular disease in men and women

Outcome	Childhood maltreatment	Men		Women		p-value for sex interaction
		Unadjusted HR (95% CI)	Adjusted HR (95% CI)	Unadjusted HR (95% CI)	Adjusted HR (95% CI)	
Cardiovascular disease	Physical abuse	1.26 (1.19; 1.34)	1.12 (1.05; 1.19)	1.39 (1.30; 1.48)	1.17 (1.10; 1.25)	0.213
	Sexual abuse	1.15 (1.04; 1.27)	1.14 (1.03; 1.26)	1.20 (1.11; 1.29)	1.09 (1.01; 1.18)	0.496
	Emotional abuse	1.36 (1.26; 1.46)	1.19 (1.10; 1.28)	1.46 (1.37; 1.55)	1.27 (1.19; 1.35)	0.121
	Emotional neglect	1.15 (1.08; 1.22)	1.09 (1.03; 1.16)	1.26 (1.19; 1.34)	1.20 (1.14; 1.28)	0.016
	Physical neglect	1.05 (0.98; 1.12)	1.17 (1.09; 1.25)	1.05 (0.99; 1.12)	1.18 (1.11; 1.25)	0.807

Adjusted for age, year of birth, ethnicity, maternal smoking, number of siblings, and family history of cardiovascular disease

* p-value corresponds to p-value for sex interaction in the adjusted model

Supplementary Table S4. Unadjusted and adjusted association between childhood maltreatment and potential mediators

Mediators	Childhood maltreatment	Men		Women		p-value for sex interaction
		Unadjusted	Adjusted	Unadjusted	Adjusted	
Continuous mediators		β (95% CI)	β (95% CI)	β (95% CI)	β (95% CI)	
Body mass index (kg/m^2)	Physical abuse	0.63 (0.54; 0.71)	0.53 (0.44; 0.62)	0.75 (0.65; 0.85)	0.69 (0.59; 0.79)	0.063
	Sexual abuse	0.24 (0.08; 0.39)	0.17 (0.02; 0.32)	0.64 (0.53; 0.76)	0.57 (0.45; 0.68)	<0.001
	Emotional abuse	0.43 (0.32; 0.53)	0.34 (0.24; 0.45)	0.40 (0.31; 0.50)	0.36 (0.27; 0.46)	0.726
	Emotional neglect	0.14 (0.06; 0.23)	0.06 (-0.02; 0.15)	0.30 (0.22; 0.39)	0.23 (0.15; 0.32)	0.021
	Physical neglect	0.45 (0.35; 0.56)	0.41 (0.31; 0.51)	0.43 (0.33; 0.53)	0.35 (0.25; 0.44)	0.761
C-reactive protein (mg/L)	Physical abuse	1.05 (1.03; 1.08)	1.06 (1.03; 1.08)	1.08 (1.05; 1.10)	1.10 (1.08; 1.13)	0.041
	Sexual abuse	1.05 (1.01; 1.10)	1.04 (0.99; 1.08)	1.07 (1.04; 1.10)	1.08 (1.05; 1.11)	0.131
	Emotional abuse	1.06 (1.03; 1.09)	1.07 (1.04; 1.10)	1.02 (1.00; 1.05)	1.04 (1.02; 1.07)	0.074
	Emotional neglect	1.01 (0.99; 1.04)	1.01 (0.99; 1.04)	1.04 (1.02; 1.06)	1.04 (1.01; 1.06)	0.223
	Physical neglect	1.10 (1.07; 1.13)	1.07 (1.04; 1.10)	1.13 (1.10; 1.15)	1.09 (1.06; 1.11)	0.158
Binary mediators		OR (95% CI)	OR (95% CI)	OR (95% CI)	OR (95% CI)	
Ever smoking	Physical abuse	1.29 (1.23; 1.36)	1.34 (1.27; 1.41)	1.42 (1.35; 1.48)	1.47 (1.40; 1.54)	0.001
	Sexual abuse	1.67 (1.52; 1.84)	1.67 (1.52; 1.84)	1.49 (1.41; 1.57)	1.52 (1.43; 1.60)	0.077
	Emotional abuse	1.40 (1.31; 1.49)	1.46 (1.37; 1.56)	1.50 (1.44; 1.57)	1.54 (1.48; 1.61)	0.078
	Emotional neglect	1.23 (1.17; 1.29)	1.24 (1.18; 1.31)	1.37 (1.32; 1.43)	1.38 (1.32; 1.44)	0.001
	Physical neglect	1.02 (0.97; 1.09)	0.99 (0.93; 1.05)	1.10 (1.05; 1.15)	1.09 (1.04; 1.14)	0.103
Anxiety/depression	Physical abuse	1.32 (1.25; 1.39)	1.32 (1.25; 1.39)	1.63 (1.56; 1.70)	1.62 (1.55; 1.69)	<0.001
	Sexual abuse	1.64 (1.49; 1.79)	1.65 (1.50; 1.80)	1.69 (1.60; 1.78)	1.67 (1.59; 1.77)	0.620
	Emotional abuse	1.98 (1.86; 2.11)	1.99 (1.87; 2.12)	2.07 (1.98; 2.16)	2.06 (1.97; 2.15)	0.240
	Emotional neglect	1.81 (1.72; 1.91)	1.83 (1.73; 1.93)	2.20 (2.12; 2.29)	2.21 (2.12; 2.30)	<0.001
	Physical neglect	1.22 (1.14; 1.30)	1.26 (1.18; 1.34)	1.35 (1.29; 1.41)	1.38 (1.32; 1.44)	0.015

Adjusted for age, year of birth, ethnicity, maternal smoking, number of siblings, and family history of cardiovascular disease

Supplementary Table S5. Unadjusted and adjusted association between the mediators and incident cardiovascular disease in men and women

Outcome	Mediator	Men		Women		p-value sex interaction
		Unadjusted	Adjusted	Unadjusted	Adjusted	
		HR (95%CI)		HR (95%CI)		
Cardiovascular disease	Body mass index	1.06 (1.06; 1.07)	1.05 (1.05; 1.06)	1.05 (1.05; 1.06)	1.05 (1.04; 1.05)	0.430
	Smoking	1.04 (0.99; 1.09)	1.12 (1.06; 1.18)	1.02 (0.97; 1.07)	1.05 (1.00; 1.11)	0.090
	Anxiety/depression	1.22 (1.15; 1.29)	1.16 (1.10; 1.23)	1.38 (1.31; 1.45)	1.31 (1.25; 1.38)	0.002
	C-reactive protein	1.10 (1.08; 1.13)	1.14 (1.11; 1.17)	1.13 (1.10; 1.16)	1.17 (1.15; 1.20)	0.159

Adjusted for age, year of birth, ethnicity, maternal smoking, number of siblings, family history of cardiovascular disease, education, and Townsend deprivation index

Supplementary Table S6. Estimated odds ratios (OR) for the association between childhood maltreatment and cardiovascular disease in men (sensitivity analysis)

CVD	Maltreatment	Mediators	TCE OR (95% CI)	NDE OR (95% CI)	NIE OR (95% CI)	Proportion mediated	Additional contribution (%)
Any CVD	Physical abuse	BMI	1.13 (1.06; 1.21)	1.10 (1.03; 1.17)	1.03 (1.02; 1.04)	24.9	
		+ Anxiety/depression	1.13 (1.06; 1.21)	1.09 (1.02; 1.16)	1.04 (1.03; 1.05)	32.9	8.0
		+ Smoking	1.13 (1.06; 1.21)	1.08 (1.01; 1.16)	1.05 (1.03; 1.06)	38.6	5.7
		+ CRP	1.13 (1.06; 1.21)	1.08 (1.01; 1.15)	1.05 (1.03; 1.06)	38.8	0.2
	Sexual abuse	BMI	1.16 (1.04; 1.30)	1.15 (1.03; 1.28)	1.02 (1.00; 1.03)	11.7	
		+ Anxiety/depression	1.16 (1.04; 1.30)	1.12 (1.00; 1.25)	1.04 (1.02; 1.07)	30.2	18.6
		+ Smoking	1.16 (1.04; 1.30)	1.12 (1.00; 1.25)	1.05 (1.01; 1.09)	31.5	1.3
		+ CRP	1.16 (1.04; 1.30)	1.10 (0.98; 1.24)	1.05 (1.01; 1.09)	34.3	2.8
	Emotional abuse	BMI	1.19 (1.10; 1.29)	1.17 (1.07; 1.27)	1.02 (1.01; 1.03)	12.3	
		+ Anxiety/depression	1.19 (1.10; 1.29)	1.14 (1.05; 1.23)	1.05 (1.02; 1.07)	27.4	15.1
		+ Smoking	1.19 (1.10; 1.29)	1.13 (1.04; 1.23)	1.06 (1.03; 1.08)	32.3	4.9
		+ CRP	1.19 (1.10; 1.29)	1.12 (1.03; 1.22)	1.06 (1.03; 1.08)	34.0	1.6
	Emotional neglect	BMI	1.10 (1.03; 1.17)	1.09 (1.02; 1.16)	1.01 (1.00; 1.02)	9.1	
		+ Anxiety/depression	1.10 (1.03; 1.17)	1.08 (1.01; 1.15)	1.02 (1.00; 1.04)	22.6	13.5
		+ Smoking	1.10 (1.03; 1.17)	1.08 (1.01; 1.15)	1.02 (1.01; 1.04)	25.9	3.3
		+ CRP	1.10 (1.03; 1.17)	1.07 (1.00; 1.14)	1.02 (1.01; 1.04)	26.5	0.7
	Physical neglect	BMI	1.18 (1.10; 1.27)	1.14 (1.06; 1.23)	1.04 (1.02; 1.05)	23.5	
		+ Anxiety/depression	1.18 (1.10; 1.27)	1.13 (1.05; 1.22)	1.04 (1.03; 1.06)	27.3	3.8
		+ Smoking	1.18 (1.10; 1.27)	1.14 (1.06; 1.22)	1.04 (1.03; 1.06)	25.8	-1.5
		+ CRP	1.18 (1.10; 1.27)	1.12 (1.04; 1.21)	1.04 (1.03; 1.06)	28.5	2.7

BMI: body mass index; CRP: C-reactive protein; NDE: natural direct effect; NIE: natural indirect effect; TCE: total causal effect

Adjusted for age, year of birth, ethnicity, maternal smoking, number of siblings and family history of cardiovascular disease as baseline confounders, and for education and Townsend deprivation index as intermediate confounders

Supplementary Table S7. Estimated odds ratios (OR) for the association between childhood maltreatment and cardiovascular disease in women (sensitivity analysis)

CVD	Maltreatment	Mediators	TCE OR (95% CI)	NDE OR (95% CI)	NIE OR (95% CI)	Proportion mediated	Additional contribution (%)
Any CVD	Physical abuse	BMI	1.16 (1.09; 1.24)	1.12 (1.05; 1.20)	1.04 (1.03; 1.05)	27.0	
		+ Anxiety/Depression	1.16 (1.09; 1.24)	1.08 (1.00; 1.16)	1.08 (1.06; 1.10)	53.1	26.1
		+ Smoking	1.16 (1.09; 1.24)	1.08 (1.00; 1.16)	1.07 (1.05; 1.10)	50.6	-2.5
		+ CRP	1.16 (1.09; 1.24)	1.07 (1.00; 1.15)	1.08 (1.06; 1.10)	55.0	4.4
	Sexual abuse	BMI	1.09 (1.00; 1.18)	1.05 (0.97; 1.14)	1.03 (1.02; 1.05)	41.0	
		+ Anxiety/Depression	1.09 (1.00; 1.18)	1.02 (0.93; 1.11)	1.07 (1.04; 1.09)	79.2	38.2
		+ Smoking	1.09 (1.00; 1.18)	1.02 (0.93; 1.11)	1.07 (1.04; 1.10)	80.3	1.2
		+ CRP	1.09 (1.00; 1.18)	1.01 (0.92; 1.10)	1.07 (1.04; 1.10)	89.2	8.9
	Emotional abuse	BMI	1.27 (1.19; 1.36)	1.24 (1.16; 1.32)	1.02 (1.02; 1.03)	10.8	
		+ Anxiety/Depression	1.27 (1.19; 1.36)	1.18 (1.10; 1.26)	1.08 (1.06; 1.10)	34.3	23.5
		+ Smoking	1.27 (1.19; 1.36)	1.19 (1.10; 1.28)	1.07 (1.04; 1.09)	29.5	-4.8
		+ CRP	1.27 (1.19; 1.36)	1.17 (1.09; 1.26)	1.07 (1.04; 1.10)	32.5	3.0
	Emotional neglect	BMI	1.20 (1.13; 1.28)	1.19 (1.12; 1.26)	1.01 (1.01; 1.02)	7.9	
		+ Anxiety/Depression	1.20 (1.13; 1.28)	1.13 (1.06; 1.20)	1.07 (1.05; 1.09)	38.8	30.9
		+ Smoking	1.20 (1.13; 1.28)	1.12 (1.05; 1.20)	1.07 (1.05; 1.09)	38.7	-0.1
		+ CRP	1.20 (1.13; 1.28)	1.12 (1.04; 1.19)	1.07 (1.05; 1.10)	41.3	2.6
	Physical neglect	BMI	1.19 (1.12; 1.27)	1.16 (1.09; 1.24)	1.03 (1.02; 1.04)	15.9	
		+ Anxiety/Depression	1.19 (1.12; 1.27)	1.14 (1.06; 1.21)	1.05 (1.04; 1.07)	30.7	14.8
		+ Smoking	1.19 (1.12; 1.27)	1.14 (1.07; 1.22)	1.05 (1.03; 1.06)	27.0	-3.7
		+ CRP	1.19 (1.12; 1.27)	1.13 (1.05; 1.20)	1.05 (1.04; 1.07)	32.0	5.0

BMI: body mass index; CRP: C-reactive protein; NDE: natural direct effect; NIE: natural indirect effect; TCE: total causal effect

Adjusted for age, year of birth, ethnicity, maternal smoking, number of siblings and family history of cardiovascular disease as baseline confounders, and for education and Townsend deprivation index as intermediate confounders

Supplementary Table S8. Comparison of cardiovascular disease (CVD) risk factors in participants with prevalent CVD at baseline and incident CVD during follow-up

	Males		Females	
	Prevalent<br>N = 21,781	Incident<br>N = 6,393	Prevalent<br>N = 21,316	Incident<br>N = 6,418
Continuous variables: mean (SD)				
Age (years)	59.1 (6.9)	58.1 (7.2)	58.1 (7.0)	57.3 (7.4)
BMI (kg/m^2)	28.6 (4.4)	27.3 (3.9)	28.0 (5.5)	26.8 (5.0)
CRP (mg/L)	2.5 (4.3)	2.3 (3.8)	3.0 (4.7)	2.6 (4.3)
SBP (mmHg)	144.9 (17.5)	142.8 (18.0)	141.7 (19.2)	136.5 (19.6)
DBP (mmHg)	85.9 (10.2)	85.0 (10.3)	83.7 (10.2)	81.2 (10.3)
Age of CVD onset*	51.1 (8.9)	62.5 (7.5)	49.4 (10.6)	61.7 (7.8)
Categorical variables: % (95%CI)				
Smoking status				
Never smoker	31.9 (31.3, 32.6)	33.5 (32.3, 34.6)	42.9 (42.2, 43.5)	42.5 (41.2, 43.7)
Former smoker	60.4 (59.7, 61.0)	56.1 (54.9, 57.3)	51.7 (51.1, 52.4)	50.8 (49.6, 52.0)
Current smoker	7.7 (7.3, 8.0)	10.4 (9.7, 11.2)	5.4 (5.1, 5.7)	6.7 (6.1, 7.3)
Depression	29.1 (28.5, 29.7)	26.0 (24.9, 27.1)	44.5 (43.9, 45.2)	44.1 (42.9, 45.3)

BMI: body mass index; CRP: C-reactive protein; CVD: cardiovascular disease; DBP: diastolic blood pressure; SBP: systolic blood pressure; SD: standard deviation

* for CVD cases before the baseline, age of CVD onset was self-reported, whilst for incident CVD cases, age of CVD onset was calculated using information from hospital records and age at recruitment. Therefore, this might not be comparable.

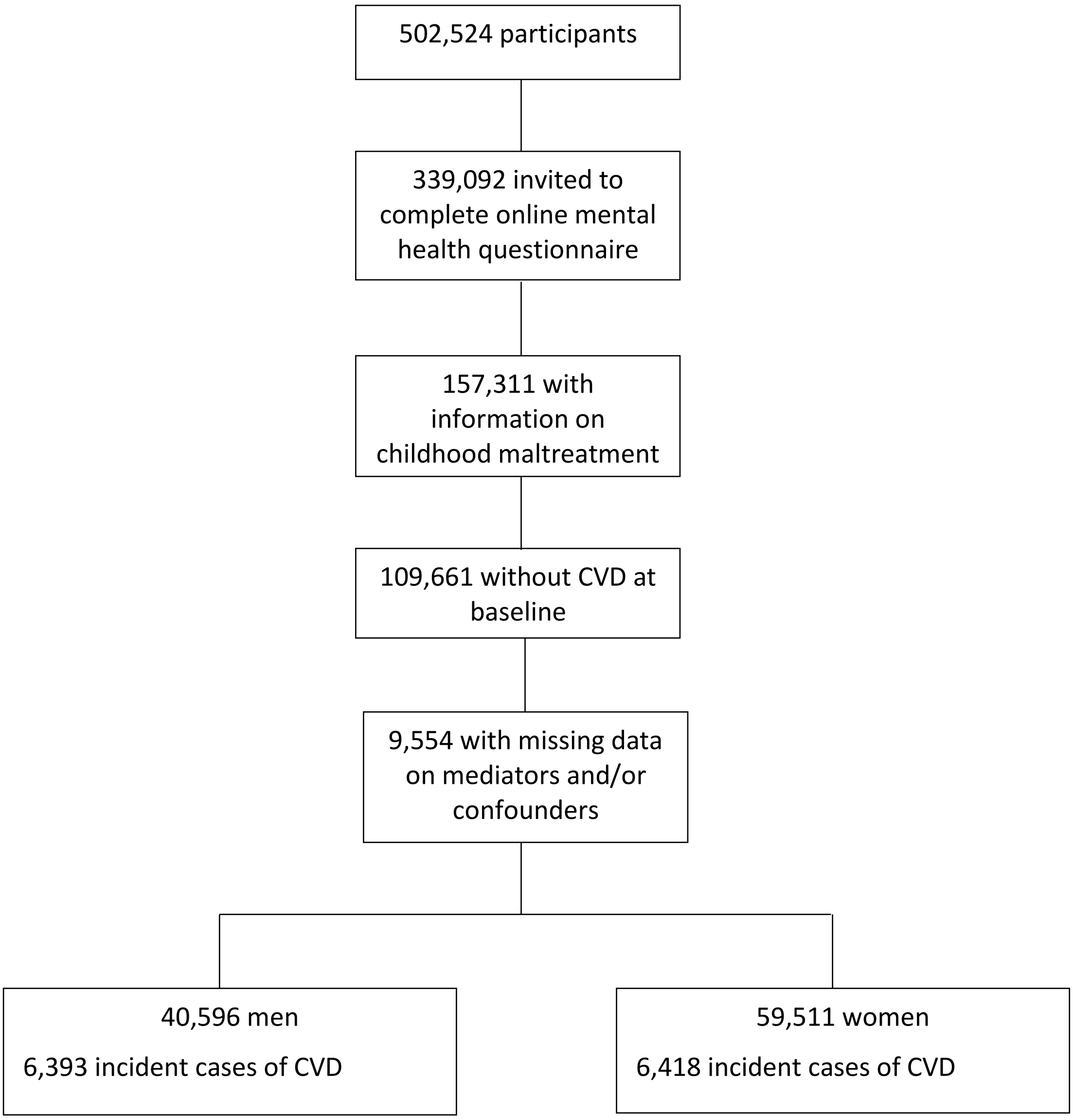

CVD: cardiovascular disease

Supplementary Figure S1. Flow diagram of participants included in the study

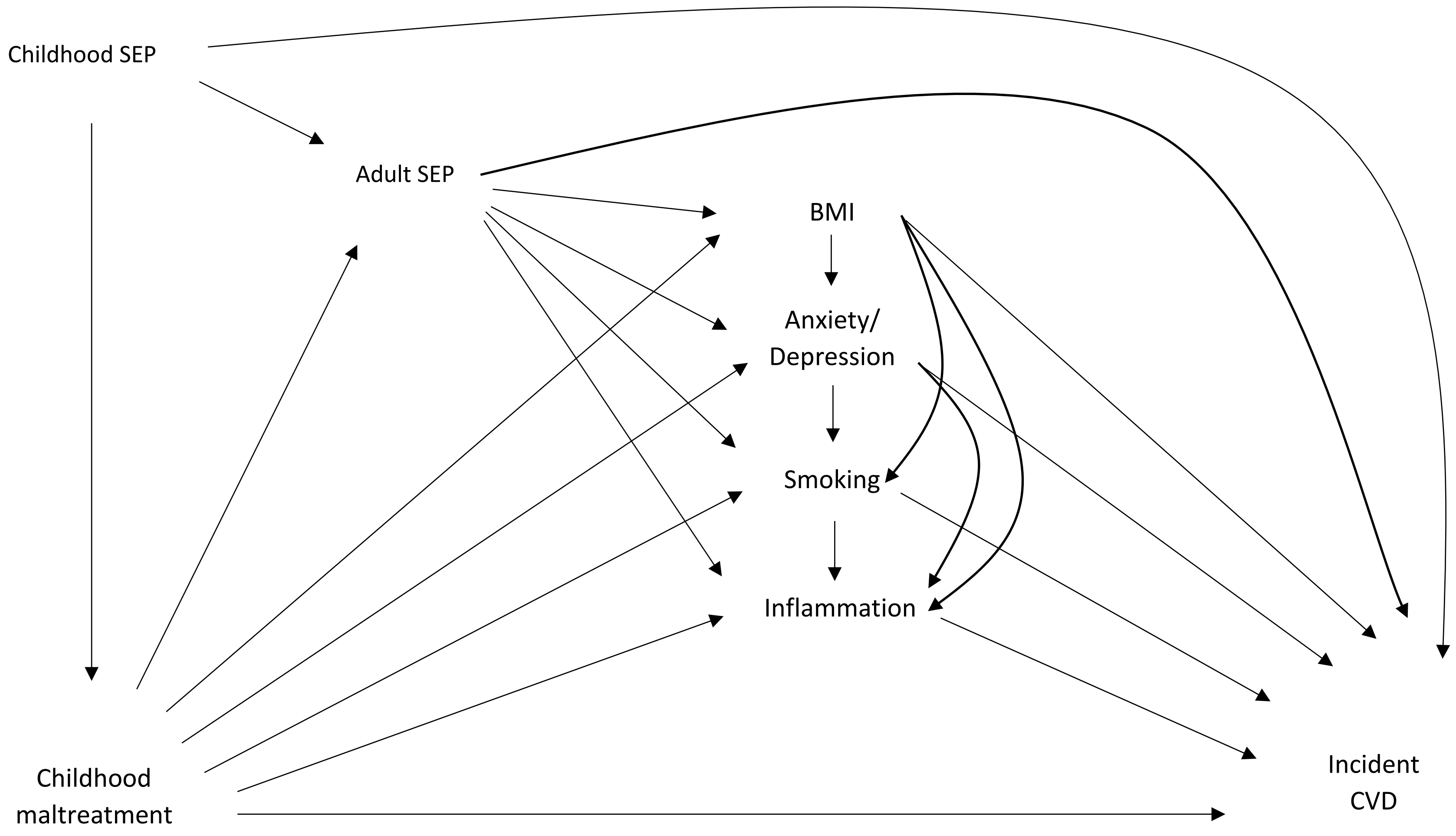

Supplementary Figure S2. Assumed causal diagram of the association between childhood maltreatment and cardiovascular disease (CVD) for sensitivity analysis

BMI: body mass index; CVD: cardiovascular disease; SEP: socioeconomic position

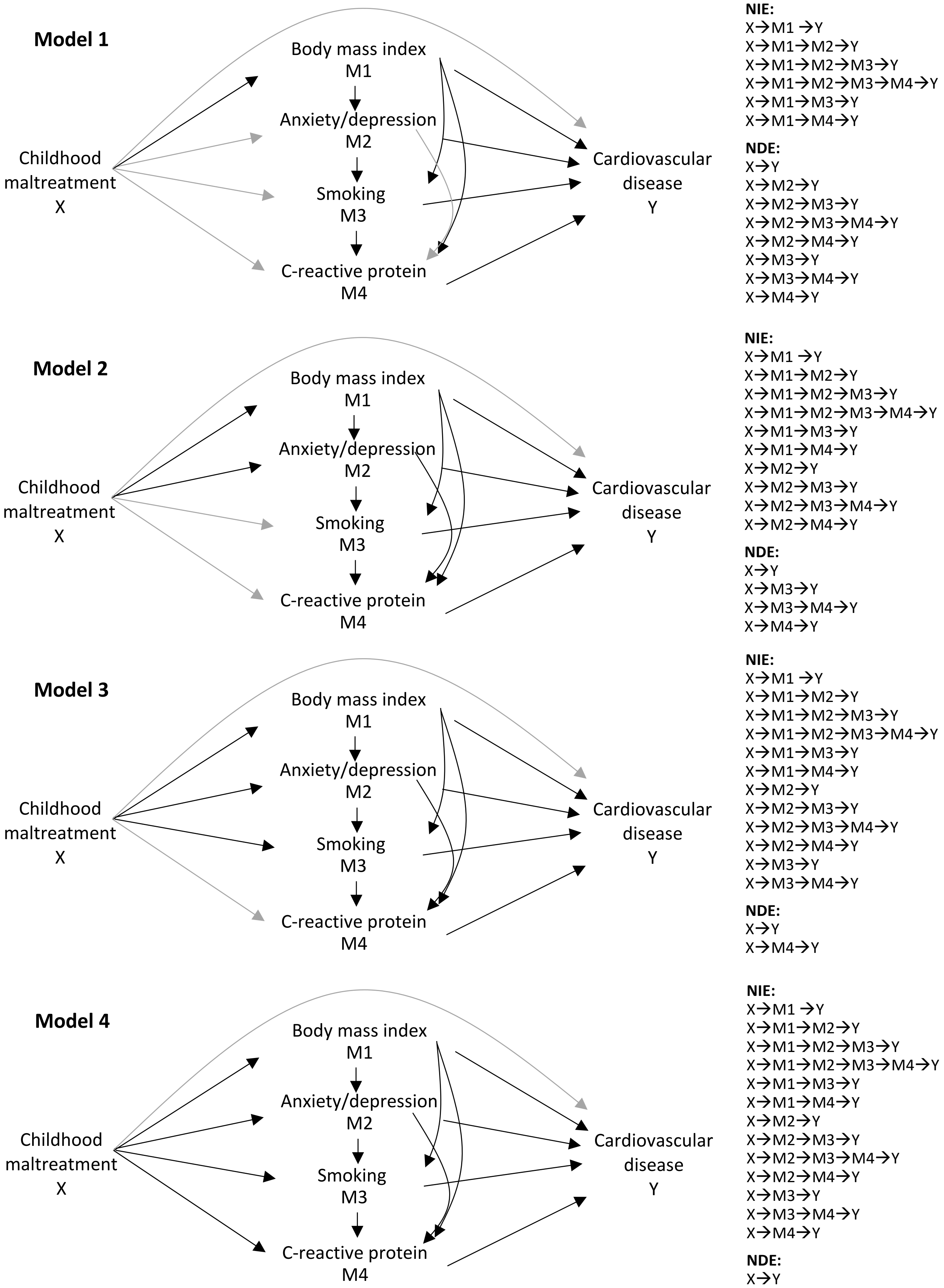

Supplementary Figure S3. Simplified causal diagrams illustrating estimated paths in Models 1-4 in the sensitivity analysis; the natural direct effect (NDE) is illustrated by the grey lines and the natural indirect effect (NIE) by the black lines.

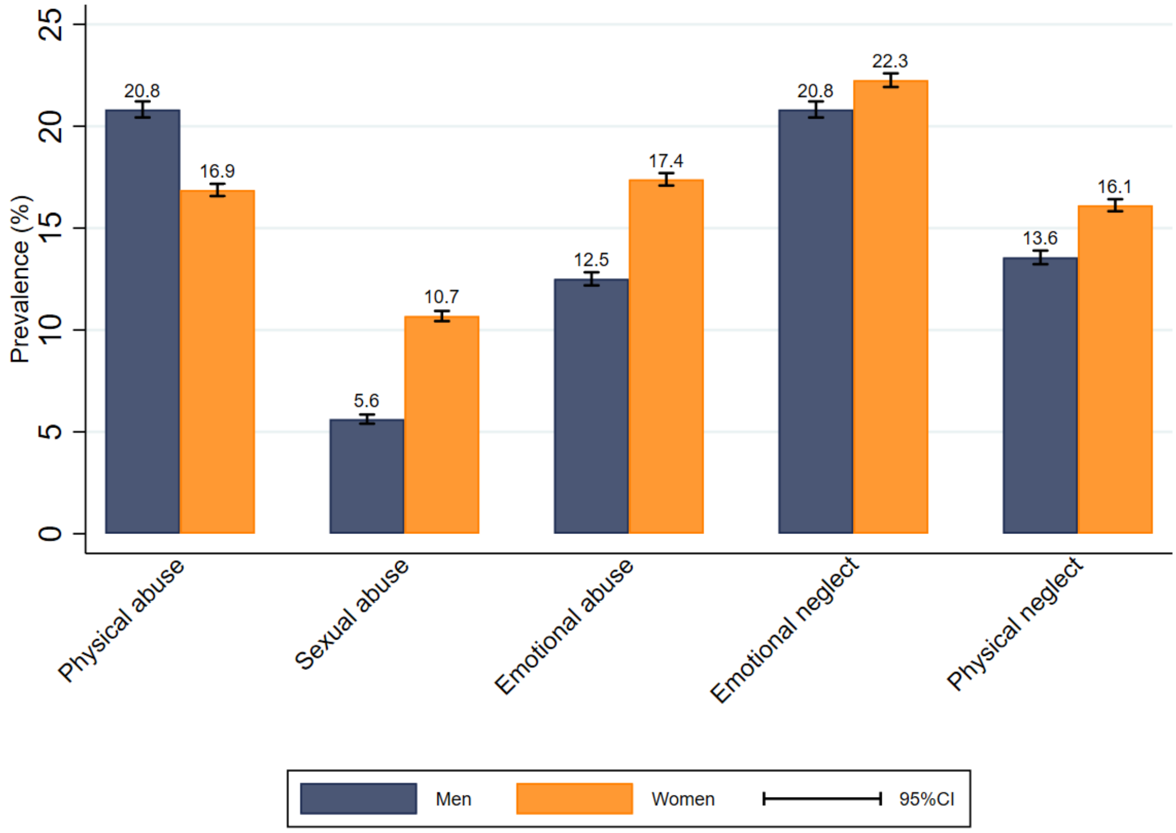

Supplementary Figure S4. Prevalence of childhood maltreatment in men and women

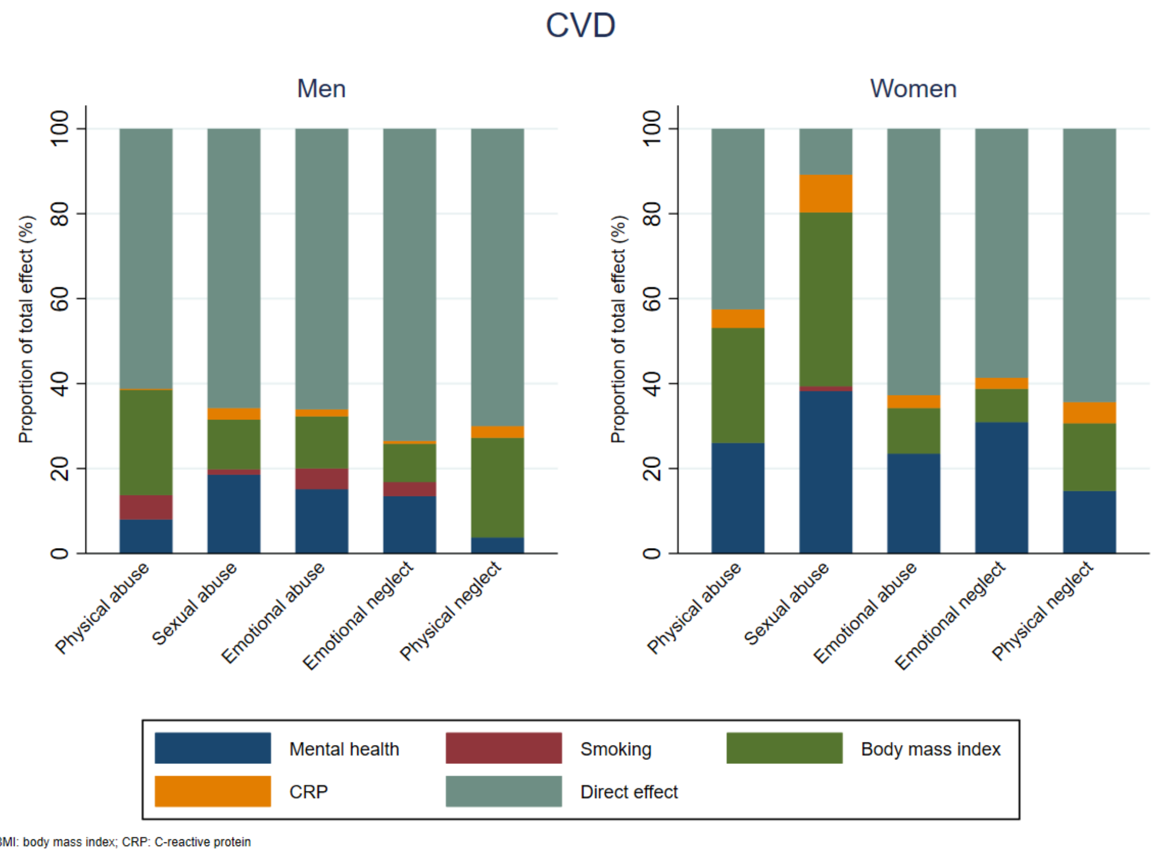

Supplementary Figure S5. Proportion mediated by mental health, smoking, body mass index and C-reactive protein in men and women in the association between childhood maltreatment and cardiovascular disease (sensitivity analysis).
